# A Comprehensive Analysis of the DUF4228 Gene Family in *Gossypium* Reveals the Role of *GhDUF4228-67* in Salt Tolerance

**DOI:** 10.3390/ijms232113542

**Published:** 2022-11-04

**Authors:** Xiaoyan Lv, Fei Wei, Boying Lian, Guo Yin, Mengxi Sun, Pengyun Chen, Li An, Hongliang Jian, Hantao Wang, Xiaokang Fu, Liang Ma, Jianhua Lu, Baoquan Wang, Hengling Wei

**Affiliations:** 1 State Key Laboratory of Cotton Biology, Institute of Cotton Research of Chinese Academy of Agricultural Sciences, Anyang 455000, China; 2 Zhengzhou Research Base, School of Agricultural Sciences, Zhengzhou University Research Base, Zhengzhou University, Zhengzhou 450001, China; 3 Handan Academy of Agricultural Sciences, Handan 056000, China

**Keywords:** *Gossypium*, domains of unknown function 4228 (*DUF4228*), VIGS, salt stress

## Abstract

Soil salinization conditions seriously restrict cotton yield and quality. Related studies have shown that the DUF4228 proteins are pivotal in plant resistance to abiotic stress. However, there has been no systematic identification and analysis of the DUF4228 gene family in cotton and their role in abiotic stress. In this study, a total of 308 *DUF4228* genes were identified in four *Gossypium* species, which were divided into five subfamilies. Gene structure and protein motifs analysis showed that the GhDUF4228 proteins were conserved in each subfamily. In addition, whole genome duplication (WGD) events and allopolyploidization might play an essential role in the expansion of the *DUF4228* genes. Besides, many stress-responsive (MYB, MYC) and hormone-responsive (ABA, MeJA) related *cis*-elements were detected in the promoters of the *DUF4228* genes. The qRT-PCR results showed that *GhDUF4228* genes might be involved in the response to abiotic stress. VIGS assays and the measurement of relative water content (RWC), Proline content, POD activity, and malondialdehyde (MDA) content indicated that *GhDUF4228-67* might be a positive regulator of cotton response to salt stress. The results in this study systematically characterized the *DUF4228s* in *Gossypium* species and will provide helpful information to further research the role of *DUF4228s* in salt tolerance.

## 1. Introduction

As one of the most severe global environmental challenges caused by climate change and irrigation practices, soil salinization is a significant factor that threatens agricultural productivity worldwide [1,2]. Around 20% of agricultural land is increasingly affected by salinity [3]. The high concentrations of Na+ and Cl^-^ in the saline–alkali soil solution will prevent the plant roots from absorbing water, and when the accumulation of Na+ and Cl− reaches a certain level, it will cause ion toxicity, a decrease in photosynthesis and respiration rates, and the accumulation of reactive oxygen species (ROS) [4,5,6,7]. When the excess radicals are not scavenged in a timely manner by antioxidant enzymes such as superoxide dismutase (SOD), peroxidase (POD), and catalase (CAT), it will cause lipid peroxidation in cell membranes, which can damage the structure and function of the membrane system, and eventually lead to irreversible metabolic dysfunction and cell death [8].

Currently, 6565 families have been annotated as Domains of unknown function (DUF), named using a combination of “DUF” and a number, such as DUF1 and DUF2 [9,10,11]. As one of the above families, the DUF4228 gene family (Pfam accession: 14009) members have been widely detected in the plant genome [12,13]. Multiple studies have revealed that DUF4228 family members are closely related to the abiotic stress response of plants. By analyzing the expression patterns of *AtDUF4228* under drought, salt, cold, and osmotic stress treatments, *AT1G10530*, *AT1G21010*, and *AT1G28190* showed the most significant changes in expression, and it is speculated that *AtDUF4228* may be involved in abiotic stress [12]. Ectopic overexpression of the gene *GmDUF4228-70* could enhance the tolerance of soybean seedlings under drought and salt stress. The change of proline, MDA, H_2_O_2_, and O^2−^ has also indicated that *GmDUF4228-70* plays an important role in soybean response to stress [13]. Under the treatments of NaCl, PEG6000, abscisic acid (ABA), and gibberellin (GA), the expression of *MsDUF* was significantly down-regulated, indicating that the *MsDUF* may play a negative regulatory role in coping with abiotic stress [14]. Recent studies have proved that genes with the DUF4228 domain are the backbone of plant resistance to fungal diseases [15]. However, there has been no systematic identification and analysis of the DUF4228 family in cotton (*Gossypium*).

The genus *Gossypium* contains 50 species, including four cultivated species: *G. hirsutum* [(AD)_1_, 2n = 4x = 52], *G.barbadense* [(AD)_2_, 2n = 4x = 52], *G.arboreum* (A_2_, 2n = 4x = 26), and *G.raimondii* (D_5_, 2n = 4x = 26), and more than 90% of cotton production is produced by *G. hirsutum* [16]. As one of the most important economic crops in the world, it produces fiber to supply the textile, chemical raw materials, and other strategically important industries. Cotton is also considered to be a pioneer crop for salinity tolerance, but high salt stress still restricts the growth and yield of cotton [17]. Under salt stress, the normal physiological functions and material metabolism of cotton are significantly affected, resulting in reduced yield and poorer fiber quality. Salt tolerance varies greatly among cotton species, and even among different varieties and growth stages of the same cotton species. Compared with upland cotton, island cotton has a better tolerance to common abiotic stresses. Salt tolerance among different species is often controlled by a few dominant genes. Therefore, it is indispensable to mine genes related to salt stress and further apply them in cotton breeding [18].

In this study, 308 *DUF4228* genes were identified in four *Gossypium* species, and phylogenetic, gene structure, duplication events, selective pressure, and *cis*-elements were further analyzed. The bioinformatics results showed that the DUF4228 mainly expanded by the WGD and events, while their structure and function might be conserved during the evolution. The quantitative RT-PCR (qRT-PCR) showed some *GhDUF4228* genes responding to the drought and salt stress, as well as ABA and methyl Jasmonate (MeJA) hormone treatments. Based on the above analyses, *GhDUF4228-67* was chosen to perform the VIGS experiment. Under salt stress, the *GhDUF4228-67*-silenced plants showed obvious wilting compare with the control plants and the relative content of the MDA and proline also determined that *GhDUF4228-67*-silenced plants were more sensitive to salt stress than control plants. Together, these results lay a valuable foundation for further studies of *GhDUF4228* genes.

## 2. Results

### 2.1. Genome-Wide Identification and Phylogenetic Analysis of DUF4228 Family Members

In total, 103, 102, 53, and 50 DUF4228 proteins were identified in *G. hirsutum*, *G.barbadense*, *G. arboretum,* and *G. raimondii*, respectively. Based on their locations on the chromosome, the members of *DUF4228s* in the chosen cotton species were named from *GhDUF4228-1* to *GhDUF4228-103*, *GbDUF4228-1* to *GbDUF4228-102*, *GrDUF4228-1* to *GrDUF4228-50*, and *GaDUF4228-1* to *GaDUF4228-53* (Table S1). The members of the tetraploid cotton species were almost twice that of the diploid cotton species. The lengths of the 308 DUF4228 proteins in cotton ranged from 121 amino acids (AA) to 615 AA. No intron was detected in most DUF4228, and only a few genes contained introns (Table S1). The Isoelectric Points (pIs) of the DUF4228 proteins in cotton were mainly distributed between 7 and 10, and the relative molecular weights (MWs) were mainly distributed between 17 and 23kDa. The GRAVY (Grand average of hydropathicity) was almost all <0 (97%), which indicates all DUF4228 proteins are hydrophilic.

**Figure 1:**
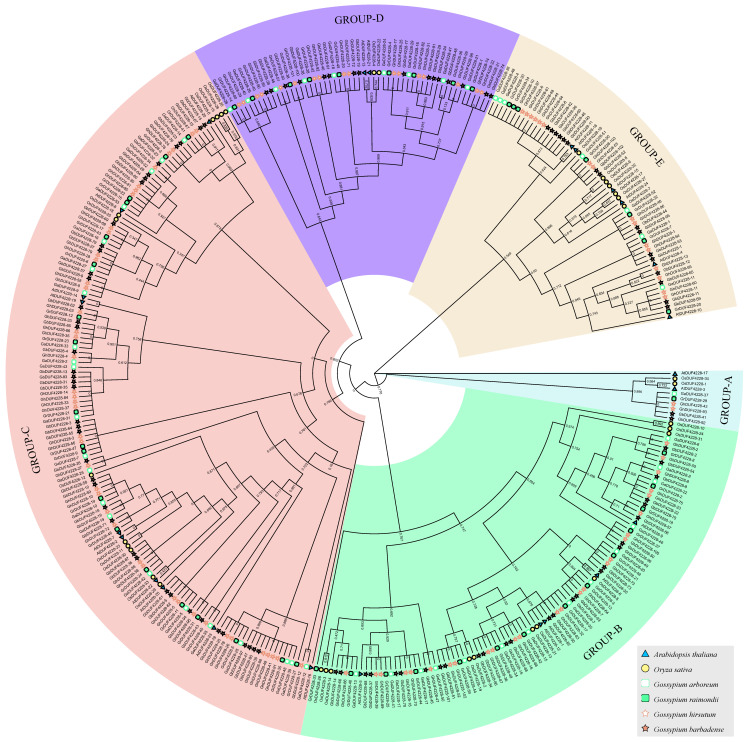
Phylogenetic relationship of DUF4228 proteins from six plant species. A phylogenetic tree was constructed using FastTree from 367 DUF4228 proteins from *Arabidopsis thaliana*, *Oryza sativa*, *Gossypium hirsutum*, *Gossypium barbadense*, *Gossypium arboretum,* and *Gossypium raimondii,* and the phylogenetic tree was divided into five subfamilies. The five subfamilies are represented by different background colors, and the six species are represented by different traits and colors.

Combined with the DUF4228 proteins from the previous study, a total of 367 proteins were constructed into a phylogenetic tree. These DUF4228 proteins were divided into five subfamilies, named GROUP-A, GROUP-B, GROUP-C, GROUP-D, and GROUP-E, respectively (Figure 1). Among them, the GROUP-C was the largest subfamily, possessing 39.7% (146) of the members, while the GROUP-A subfamily had the fewest members (10 members, 2.7%). Meanwhile, the DUF4228 proteins from six species were distributed in five subfamilies, and no species-specific subfamily was found.

### 2.2. Gene Structure, Protein Motifs, and Chromosomal Location Analysis

We detected the DUF4228 domain in all of the GhDUF4228 proteins, most of *GhDUF4228* protein sequences were almost occupied by the DUF4228 domain, and no other domains were detected (Figure S1).

A total of 20 motifs were identified in 103 *GhDUF4228* genes, named motif 1–motif 20 (Table S2). Some motifs were present in the vast majority of members, for example, 99% of GhDUF4228 proteins contained motif 1, motif 2, and motif 4. The distribution of the motifs within each subfamily was conserved. For example, GROUP-A subfamily members only contained motifs 1, 2, 4, 5, 6, and 9, while GROUP-C subfamily members mainly contained motifs 1, 2, 3, 4, and 5. However, some motifs were unique to the specific subfamily. For example, motif 3 was distributed in 86% of the proteins, but not in the GROUP-A subfamily. Motif 12, motif 13, and motif 14 only existed in the GROUP-C subfamily. Motif 15 and motif 17 existed specifically in the GROUP-B subfamily. We speculated that these results might be related to the conservation of their functions.

**Figure 2:**
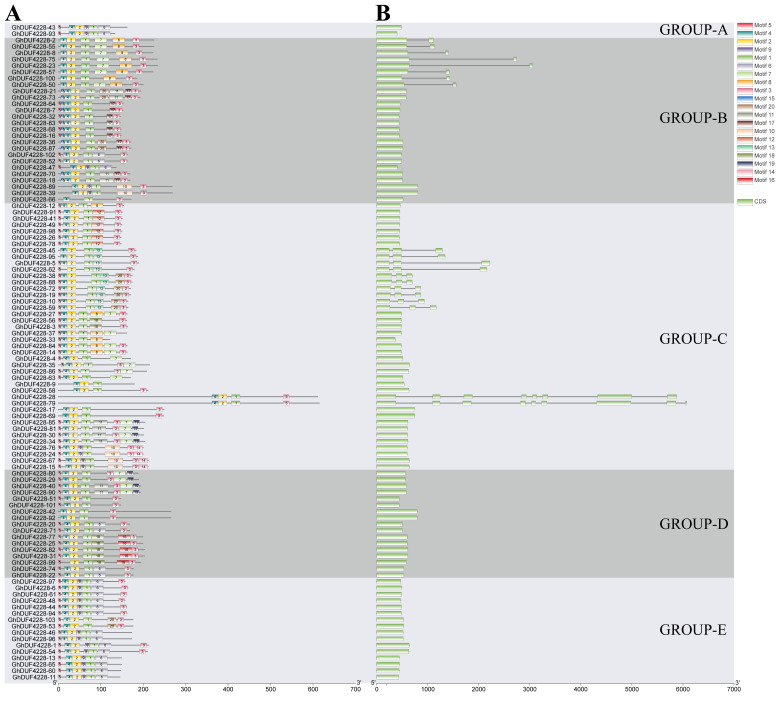
Conserved protein motifs and gene structure analysis of *GhDUF4228* genes. (**A**): Conserved motif distribution in the *GhDUF4228* genes, with 20 motifs represented by boxes with different colors. (**B**): The exons and introns of the *GhDUF4228* genes are represented by green boxes and black lines, respectively. The scale at the bottom is used to infer the length of proteins and genes.

Except for a few genes, most genes contained only one exon and no intron (Figure 2). In general, genes in the same subfamily were similar in intron number and exon length. For example, genes in GROUP-A, GROUP-D, and GROUP-E subfamily had only one exon, and their changes in exon length were also conserved. However, the *GhDUF4228-79* in the GROUP-C subfamily contained the largest number of introns, with eight introns and nine exons. This was followed by *GhDUF4228-28*, which contained seven introns and eight exons. In addition, 10 *GhDUF4228* genes (9.7%) contained 2 introns, and 8 *GhDUF4228* genes (7.7%) contained 1 intron. Analysis of gene structure and protein motifs further supported the phylogenetic relationship of *DUF4228s*.

**Figure 3:**
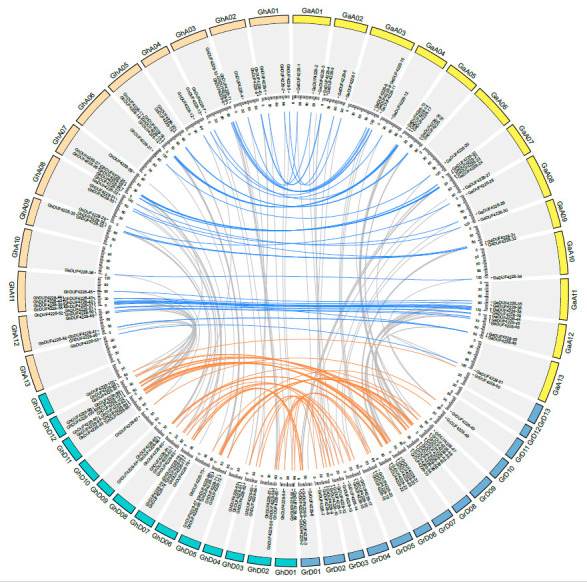
Chromosome distribution and collinearity of duplicated gene pairs of DUF4228 genes. Chromosomes are represented by differently colored boxes, distributed in the outer circle. Collinear gene pairs between *GhDUF4228* genes and *GaDUF4228* genes, between *GhDUF4228* and *GrDUF4228* genes, are represented by blue and orange lines, respectively.

*DUF4228* genes of the four *Gossypium* species were unevenly distributed on the chromosomes (Figure 3; Table S1). Except for the unclear chromosomal locations of *GaDUF4228-52* and *GaDUF4228-53*, the *DUF4228* genes of the four *Gossypium* species could be mapped to specific chromosomes, indicating that the *DUF4228* genes have evolved maturely [19]. For *G. raimondii* genome, the DUF4228 genes were mainly distributed on chromosomes 01, 07, 08, and 09, but not on chromosome 12. Moreover, *DUF4228* genes from *G.arboreum* were mainly distributed on chromosomes 05, 07, 09, and 12. In *G. hirsutum* and *G. barbadense*, *DUF4228* genes were mainly distributed on chromosomes 05, 07, 11, and 12 of the At subgenome and Dt subgenome. The distribution of *DUF4228* genes in the *G.arboreum* and At subgenome of *G. hirsutum* was highly consistent, while the distribution of *G. raimondii* was slightly distinct from that of the Dt subgenome of *G. hirsutum*.

### 2.3. Gene Duplication and Selection Pressure Analysis of DUF4228 Genes

**Figure 4:**
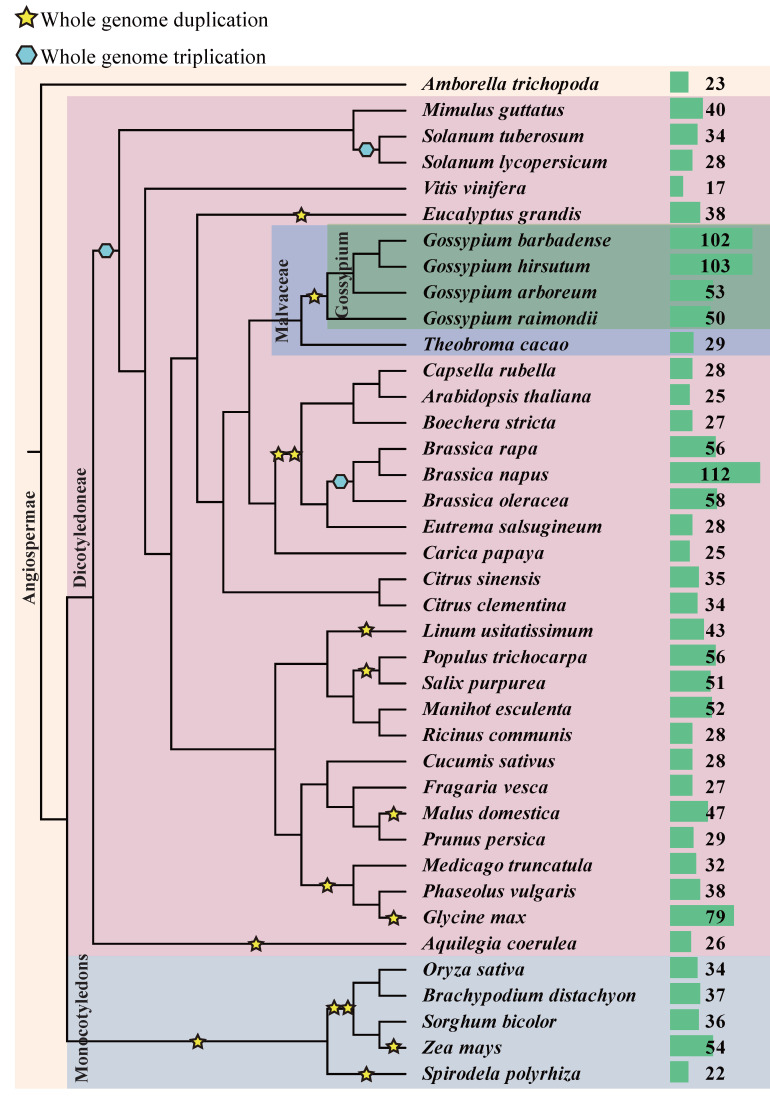
The phylogeny of 39 plants used in this study and the quantity of identified DUF4228 proteins. The right side shows the amount of DUF4228 protein detected in each plant. Whole genome duplication and whole genome triplication are represented by pentagram and hexagon symbols, respectively. The order of branch and divergence time was retrieved from the Timetree database (http://www.timetree.org/ (accessed on 12 July 2021)).

To study the expansion pattern of *DUF4228* genes during the evolution, we performed a comprehensive search in 35 other plant species and identified a total of 1689 *DUF4228* genes in 39 species (Figure 4). We found that DUF4228 was widespread in monocotyledonous and dicotyledonous plants, and the number of *DUF4228* genes varied greatly in different plants. Two diploid cotton species *G. arboreum* and *G. raimondii* experienced a whole genome duplication (WGD) event compared to *Theobroma cacao*, the number of *DUF4228* genes in *T. cacao* was 29, and the number of *DUF4228* genes in two diploid cotton species was about 1.7 times that of *T. cacao*. Similarly, *Brassica rapa* and *Brassica oleracea* experienced one WGD event compared to *A. thaliana*, and the number of *DUF4228* genes was almost twice that of *A. thaliana*.

Next, we further detected the duplication type of *DUF4228* genes by MCScanX. In the four *Gossypium* species, the WGD event accounted for more than 60%, followed by the dispersed duplication event, which accounted for about 30%, and a small amount of proximal, singleton, and tandem duplication events (Table S3). Therefore, we believe that the expansion of the cotton DUF4228 gene family is mainly affected by the WGD event. On the other hand, the expansion of the DUF4228 gene family was also affected by allopolyploidization in some plants. We found that the number of *DUF4228* genes in the allotetraploid cotton species *G. hirsutum* and *G. barbadense* was almost the sum of that of *G. arboreum* and *G. raimondii* (Figure 4). The diploid *DUF4228* genes were relatively conserved after allopolyploidization. Similarly, the number of the DUF4228 in the allotetraploid *Brassica napus* was almost the sum of *B. rapa* and *B. oleracea*. Therefore, WGD event and allopolyploidization may play a very important role in the expansion of the *DUF4228* genes.

The Ka/Ks ratios between the 218 duplicated gene pairs from four *Gossypium* species were all less than 1, of which 30 pairs had a Ka/Ks value of less than 0.1, and 184 pairs (84.4%) had a Ka/Ks value in the range of 0.1–0.5; this suggests that the *DUF4228* genes in cotton underwent strong purifying selection during evolution (Table S4).

### 2.4. Cis-Elemental Prediction of GhDUF4228 Promoters

The *cis*-elements on the promoter not only contained a large number of core elements, such as TATA-box and CAAT-box, but also included stress-responsive elements, hormone-responsive elements, and light-responsive elements (Figure S2). The stress response elements were mainly related to drought and high salinity (MYB and MYC), heat shock response (STRE), and low temperature (AE-box). Among the 103 *GhDUF4228* genes, 102 genes contained MYB elements, and 97 genes contained MYC elements. The hormone-responsive elements mainly included ABA (ABRE) and MeJA (CGTCA-motif). In the promoter region of *GhDUF4228* genes, 78 genes had ABRE elements, and about 66 genes contained CGTCA-motif elements. Besides, there were also a large number of light-responsive elements GT1-motif and I-box, as well as ARE, which are involved in anaerobic induction. According to the prediction results, *GhDUF4228* genes may be associated with hormones and various abiotic stresses.

### 2.5. Expression Patterns of GhDUF4228 Genes in Different Tissues

**Figure 5:**
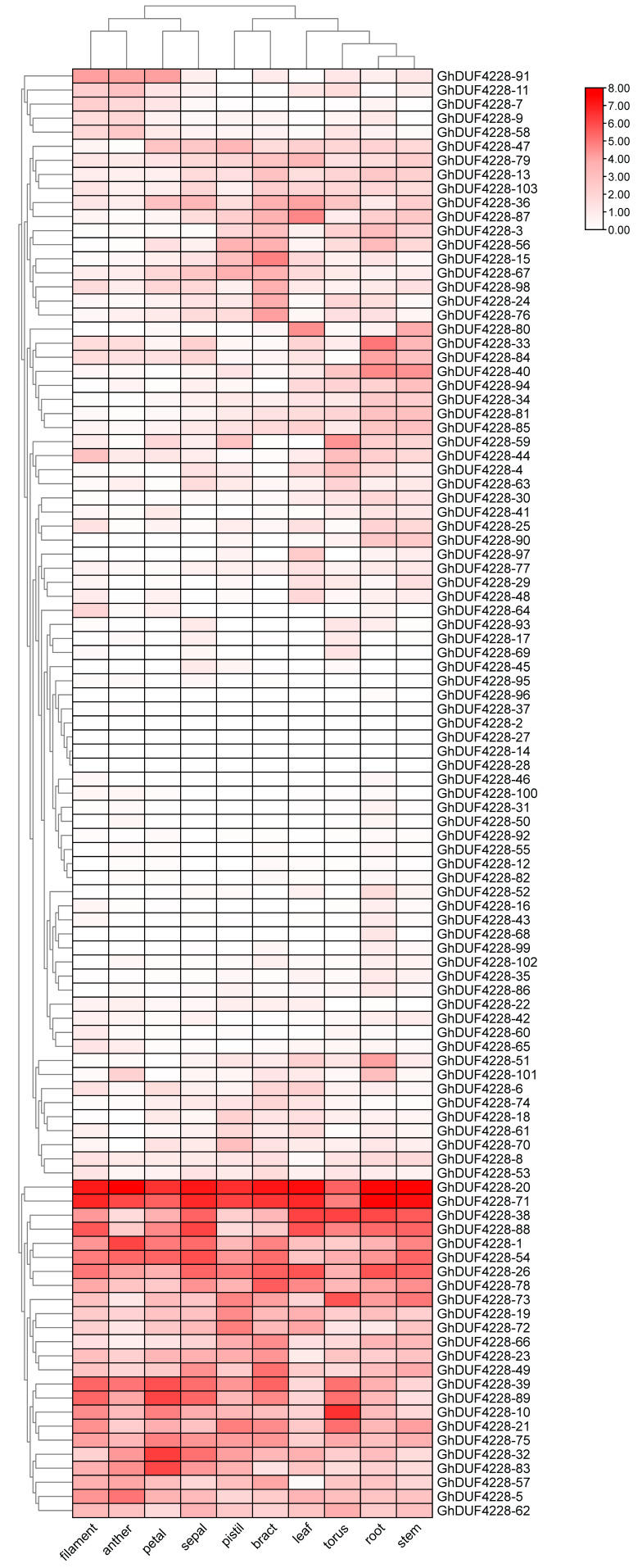
Expression profiles of *GhDUF4228* genes in different tissues of *Gossypium hirsutum*. The color scale in the upper right corner is the FPKM value, normalized by log_2_ (FPKM + 1).

Gene expression patterns at different growth stages may indicate gene functions [20], so we analyzed the expression of *GhDUF4228* genes in 10 tissues (filament, anther, petal, sepal, pistil, bract, leaf, torus, root, stem) based on RNA-seq data (Figure 5). The expression level of *GhDUF4228* genes varied among 10 tissues, and their expression pattern was distinct. Some genes were highly expressed in all 10 tissues, such as *GhDUF4228-20* and *GhDUF4228-71*. Some genes were rarely expressed in all tissues, such as *GhDUF4228-2*, *14*, *27*, *28*, *37,* and *96*. Some genes were only highly expressed in specific tissues, for example, the specific expression of *GhDUF4228-80* in stem and leaf tissues. The observations suggested that the expression of *GhDUF4228* genes in 10 tissues was inconsistent, which might be closely related to its function in different tissues.

### 2.6. Expression Patterns of GhDUF4228 Genes under Abiotic Stresses

**Figure 6:**
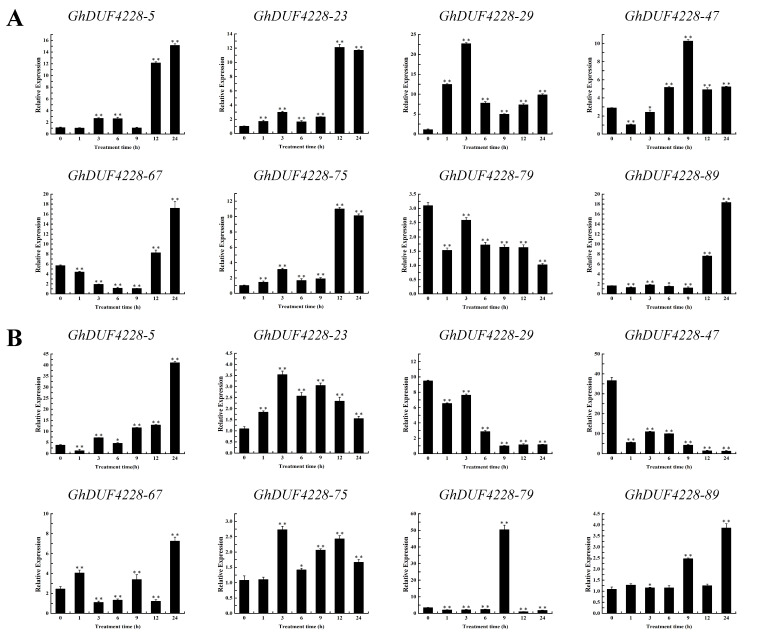
Expression patterns of *GhDUF4228* genes under salt and drought stress. (**A**) Expression levels of *GhDUF4228* genes at 0, 1, 3, 6, 9, 12, and 24 h under NaCl treatment, (**B**) expression levels of *GhDUF4228* genes at 0, 1, 3, 6, 9, 12, and 24 h under PEG treatment. Error bars represent the standard deviation of three independent biological replicates. (* p < 0.05, ** p < 0.01 Student’s *t*-test).

**Figure 7:**
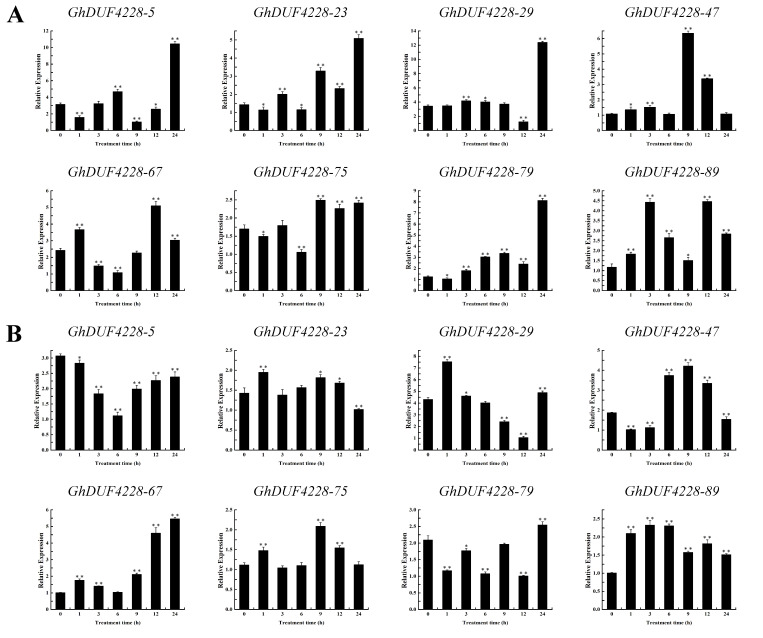
Expression patterns of *GhDUF4228* genes under ABA and MeJA treatments. (**A**) Expression levels of *GhDUF4228* genes at 0, 1, 3, 6, 9, 12, and 24 h under ABA treatment. (**B**) Expression levels of *GhDUF4228* genes at 0, 1, 3, 6, 9, 12, and 24 h under MeJA treatment. Error bars represent the standard deviation of three independent biological replicates. (* p < 0.05, ** p < 0.01 Student’s *t*-test).

Plant resistance to environmental stress is a complex process. The above results showed that *GhDUF4228* genes might play a role in various abiotic and hormones stresses. To gain a further understanding of the regulation of *GhDUF4228* genes under different environmental conditions, the expression pattern of *GhDUF4228* genes under NaCl, PEG, ABA, and MeJA treatments was detected. We found that almost all *GhDUF4228* genes responded to stress, although they did so in different ways. Based on the previous RNA-seq data, we selected eight *GhDUF4228* genes (*GhDUF4228-5*, *-23*, *-29*, *-47*, *-67*, *-75*, and *-89*) to detect their response to the NaCl and PEG treatment. Under the treatment of NaCl, the expression level of *GhDUF4228-5*, *-23*, *-29*, *-47*, *-67*, *-75*, and *-89* were significantly up-regulated, while *GhDUF4228-79* was significantly down-regulated (Figure 6A). After PEG treatment, the expression of *GhDUF4228-5*, *-23*, *-67*, *-75*, and *-89* was markedly up-regulated, and the expression of *GhDUF4228-29*, *-47*, and *-79* was strikingly down-regulated (Figure 6B). All genes were up-regulated under ABA treatment (Figure 7A). The expression levels of *GhDUF4228-29*, *-67*, *-75*, *-79*, and *-89* were all notably up-regulated, and *GhDUF4228-5*, *-23*, and *-47* were all down-regulated under MeJA treatment (Figure 7B). Besides, the changing trends of expression levels of *GhDUF4228-23* and *-75* were highly similar under the four treatments. *GhDUF4228-67*, *-75*, and *-89* were up-regulated in all four treatments, especially *GhDUF4228-67*, which not only responded positively to NaCl and PEG treatments but was also significantly up-regulated under ABA and MeJA treatments.

### 2.7. Silencing of GhDUF4228-67 Reduces Salt Tolerance in Cotton

**Figure 8:**
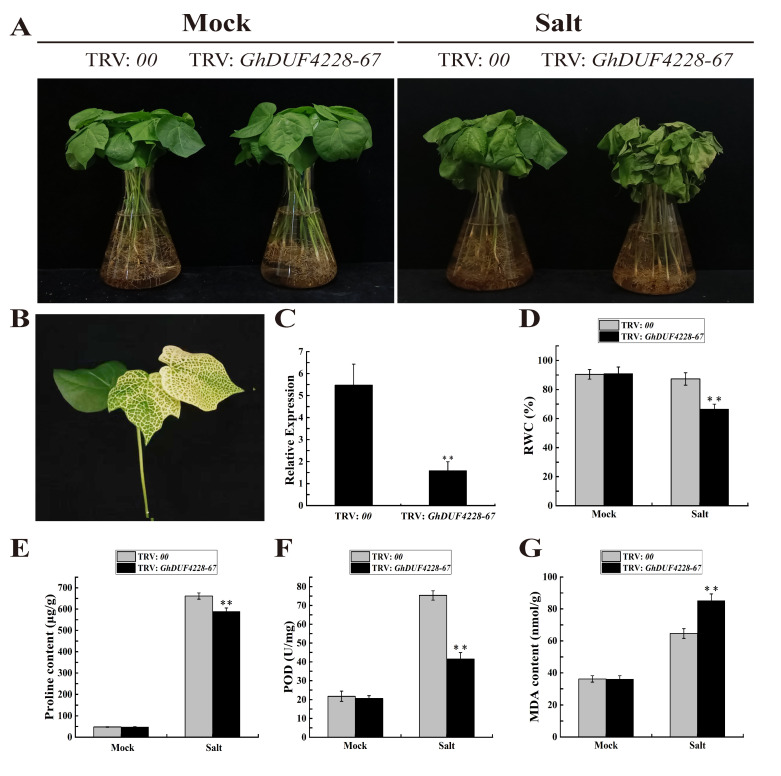
Silencing of the *GhDUF4228-67* gene reduces salt tolerance in cotton. (**A**) Phenotypes of control plants (TRV: *00*) and *GhDUF4228-67*-silenced plants (TRV: *GhDUF4228-67*) before and four days after salt stress treatment, the concentration of NaCl solution is 200 mM. (**B**) Leaf albinism of TRV: *GhPDS* (positive control). (**C**) Expression levels of *GhDUF4228-67* in control plants (TRV: *00*) and *GhDUF4228-67*-silenced plants (TRV: *GhDUF4228-67*). (**D**–**G**) Changes in relative water content (RWC), proline content, peroxidase (POD) activity, and malondialdehyde (MDA) content in control plants and *GhDUF4228-67*-silenced plants before and after salt stress treatment. Error bars represent the standard deviation of three independent biological replicates. (** p < 0.01 Student’s *t*-test).

We observed that *GhDUF4228-67* expression was induced by salt treatment. To further explore the effect of *GhDUF4228-67* under salt stress, we silenced cotton *GhDUF4228-67* by VIGS assay. When the seedlings grew to the three-leaf stage, gene-silenced and control plants were treated with 400 mM NaCl solution. After the injection after 10 days, the TRV: GhPDS plants showed an albino phenotype, indicating that the experiment was effective (Figure 8B). The qRT-PCR showed that the expression level of *GhDUF4228-67* in TRV: *GhDUF4228-67* plants was substantially lower than that in TRV: 00 plants, indicating that this gene expression was successfully suppressed (Figure 8C). After the salt treatment for 72 h, the degree of wilting and yellowing of leaves of TRV: *GhDUF4228-67* plants was dramatically higher than that of TRV: 00 plants (Figure 8A). Before the salt treatment, the RWC, proline content, POD activity, and MDA content of the TRV: 00 plants and gene-silenced plants were basically no different. After the salt treatment, the relative water content RWC of TRV: *GhDUF4228-67* gene-silenced plants decreased by 21% compared with the TRV: 00 plants (Figure 8D), and the proline content was 73 μg/g lower than that of the TRV: 00 plants (Figure 8E), the POD activity was only 1/2 of the TRV: 00 plants (Figure 8F), while the MDA content of the gene-silenced plants was 20 nm/g higher than that of the TRV: 00 plants (Figure 8G). The content of MDA indirectly reflects the damage degree to plant cells, and POD is one of the key enzymes of the enzymatic defense system of plants under adverse conditions. Generally, the proline content of plants is low, but it accumulates in large quantities when under stress, and the accumulation amount is positively correlated with the stress resistance of plants. Combined with the analysis of the above experimental results, the silencing of *GhDUF4228-67* may reduce the salt tolerance of cotton.

## 3. Discussion

Cotton is an important economic crop that can provide natural fibers for the textile industry and raw materials for industrial production [21]. Recent studies have shown that DUF4228 proteins are widely involved in the process of plant defenses against abiotic stress [12,13]. Unfortunately, no research has been reported on DUF4228 proteins in cotton. In this study, the evolutionary relationship, gene amplification, selection pressure, expression analysis, and *cis*-elements of cotton DUF4228 proteins were comprehensively and systematically analyzed. Those results showed that the expression pattern of GhDUF4228 proteins was different under various treatments, and *GhDUF4228-67* may play an important role in the process of salt stress.

### 3.1. Phylogenetic Analysis of the DUF4228 Proteins

Based on previous studies, we conducted a comprehensive analysis of the cotton DUF4228 proteins for the first time. The DUF4228 from the six species was distributed in five subfamilies (Group A–Group E). No species-specific subfamily was found, implying that the DUF4228 proteins in cotton might be relatively conserved during cotton evolution.

Motifs are short patterns retained by purification selection, and a motif may correspond to a protein binding site. Therefore, motifs are one of the basic functional units of molecular evolution [22]. In this study, a total of 20 motifs were identified in *G. hirsutum*. Combined with the phylogenetic tree, it was found that the number and location distribution of motifs in the same subfamily were similar, which suggests that the function of each subfamily might be different, while they also showed relative conservation within each subfamily. Differences in exon numbers suggest that genes may have different functions [21]. From the perspective of gene structure, cotton *DUF4228* genes have fewer introns, which is consistent with previous studies [13]. Alternative splicing is one of the major sources of proteome diversity in multicellular eukaryotes [23]. The study found that more than 80% (83) of *GhDUF4228* genes did not have introns, the low number of introns made *GhDUF4228* genes less likely to undergo alternative splicing, and there was also less variation in exon length within the same subfamily. Therefore, the structure of *GhDUF4228* genes may be highly conserved during evolution.

### 3.2. Evolution and Expansion of the DUF4228 Gene Family

During plant evolution, large-scale replication events are important for biological evolution. Gene duplication provides abundant genetic material for the emergence of new functions and is thought to enhance species diversity and environmental adaptation [24,25]. There are five main methods of gene duplication (WGD, Dispersed, Proximal, Singleton, and Tandem), among which, the gene functions generated by WGD and tandem duplication events are the slowest to differentiate in function, while other duplication patterns contribute more to evolutionary novelty [26]. Four *Gossypium* species and *T. cacao* are both members of the Malvaceae family and have a close evolutionary relationship [27]. *G. raimondii* and *G. arboreum* experienced a WGD event relative to *T. cacao*, and the number of *DUF4228* genes in the two diploid cotton species was about 1.7 times that of *T. cacao*. *B. rapa* and *B. oleracea* experienced WGD events compared to *A. thaliana*, and the number of DUF4228 proteins was more than twice that of *A. thaliana* (Figure 4). We further found that the WGD event accounted for 66.8% of all duplication events, followed by the dispersed duplication event, accounting for 31.8% (Table S3). Therefore, it was concluded that WGD is the driving force controlling the expansion of the *DUF4228* genes. On the other hand, allopolyploidization also had a strong effect on the expansion of the DUF4228 family. The quantity of *DUF4228* genes in the allotetraploid cotton species was almost the sum of the two diploid cotton species (Figure 3 and Figure 4). Similarly, the amount of *DUF4228* genes in allotetraploid species *B.napus* was almost the sum of diploid species *B. rapa* and *B. oleracea* (Figure 4). Therefore, WGD events and allopolyploidization may be the driving force controlling the expansion of the DUF4228 gene family.

Duplicated genes may undergo various functional differentiations during evolution, such as neofunctionalization, subfunctionalization, and neofunctionalization [28,29,30]. The ratio of Ka/Ks can explain the selection pressure faced by genes [28]. Ka/Ks < 1 for purification selection, Ka/Ks = 1 for neutral selection, and Ka/Ks > 1 for positive selection [31,32]. Our results showed that all of the duplicated *DUF4228* genes underwent purifying selection with limited functional differences, implying that the function of *DUF4228* genes might be highly conserved during many rounds of duplication events.

### 3.3. Potential Functional Analysis of DUF4228 Proteins

The expression of eukaryotes is regulated in many ways, and the transcriptional regulation of upstream promoters is the main mechanism of plant gene expression regulation [21]. Various *cis*-elements were detected in the promoter region of *GhDUF4228* genes, most of which included abiotic stress-responsive elements, hormone-responsive elements, and light-responsive elements. MYB recognition elements are contained in the promoters of downstream target genes such as *rd22*, *rd17,* and *rd29* related to stress resistance in *A. thaliana* [33]. Besides, MYC recognition elements could be induced by high salt, ABA, and drought [34]. In the promoter region, more than 94% of the *GhDUF4228* genes contained large amounts of MYB and MYC, which suggest *GhDUF4228* genes may play an important role in cotton response to drought and salt treatment. In addition, *GhDUF4228* genes containing ABA-responsive elements and MeJA-responsive elements accounted for 76% and 64%, respectively. MeJA and ABA not only regulate plant growth and development but also participate in plant defense responses to environmental stresses, such as mechanical damage and osmotic stress [35]. Hence, we speculated that DUF4228 proteins might play a positive regulatory role in cotton resistance to abiotic stress and are influenced by exogenous hormones.

Under the NaCl and PEG treatments, the expression levels of *GhDUF4228* genes were significantly changed and showed different trends. Therefore, they might closely relate to the process of plants responding to high salt and drought stress, which is consistent with previous findings [12,13]. The promoter of *GhDUF4228* genes contained many hormone-responsive elements, especially ABA and MeJA. These results showed that *GhDUF4228s* could respond quickly to hormone treatment. Consequently, *GhDUF4228s* responds to salt and drought stress and may be regulated by exogenous hormones ABA and MeJA. Among them, the expression patterns of *GhDUF4228-23* and *-75* were highly similar in different tissues and abiotic stress treatments. Given the types and distribution of conserved motifs of *GhDUF4228-23* and *-75* were consistent, we speculated that *GhDUF4228-23* and *-75* had similar functions. Moreover, *GhDUF4228-67* had obvious responses to the salt stress, indicating that *GhDUF4228-67* likely plays a more crucial role than other *GhDUF4228* genes in response to salt stress. The previous study also indicated its homologue *GmDUF4228-70* played a role in salt stress in soybean [13]. Therefore, we chose *GhDUF4228-67* for further research.

Next, the GhDUF4228-67 gene-silenced plants showed obvious wilting compared with control plants, which was consistent with the previous study of the *GmDUF4228-70* in soybean [13]. Furthermore, the RWC, proline content, and POD activity of the *GhDUF4228-67* gene-silenced plants were significantly lower than those of the control plants, while the MDA content was higher than that of the control plants. Proline is one of the most widely distributed metabolites in plants; its functions include regulation of osmotic potential and scavenging ROS, and it plays a very important role under abiotic stresses such as salt, drought, and high temperature [7]. Generally, the proline content of plants is low, but it accumulates in large quantities when under stress, and the accumulation amount is positively correlated with the stress resistance of plants [36,37]. When plants are subjected to salt stress, reactive oxygen species (ROS) and MDA will accumulate in large quantities, which will affect the normal growth of plants [38,39]. Moreover, studies have shown that POD is one of the key enzymes for scavenging ROS [40]. The content of MDA indirectly reflects the damage degree to plant cells [41]. Based on the above results, it can be concluded that *GhDUF4228-67* might be a positive regulator of plant resistance to salt stress.

## 4. Materials and Methods

### 4.1. Identification of DUF4228 Proteins in Cotton

We first obtained the DUF4228 proteins in *A. thaliana* and *Oryza sativa* from a previous study [12]. Next, we downloaded 37 other plant species to identify DUF4228 proteins. The detailed information on these species is listed in Table S5. Next, a Hidden Markov Model (HMM) corresponding to the DUF4228 family (PF14009) was obtained from the Pfam website (https://pfam.xfam.org/ (accessed on 9 July 2021)), to search the above genome annotation by performing the Hmmsearch program in Hmmer software (version 3.2) [9,42]. All the protein sequences were confirmed in the DUF4228 domain in Pfam database by performing the InterProScan program (version 5.51RC1-84.0) [43]. The protein properties of DUF4228 proteins were predicted by the ProtParam module in Biopython [44].

### 4.2. Phylogenetic Analysis of DUF4228 Proteins

The DUF4228 protein sequences of *A. thaliana*, *O. sativa,* and four *Gossypium* species were aligned by performing MAFFT (version 7.310) [45]. Next, BMGE was used to remove gaps in the alignment by using the BLOSUM62 matrix, and the gap rate was set as a cut-off of 50% [46]. The aligned protein was then used to construct a phylogenetic tree using FastTree (version 2.1.11) with the LG model and finally visualized on the Evolview website (http://www.evolgenius.info/evolview/ (accessed on 20 July 2021)) [47,48].

### 4.3. Gene Structure, and Motif Analysis of GhDUF4228s

The exon/intron position information in the chosen *Gossypium* species was obtained from their genomic annotation files (GFF/GTF files). The full-length protein sequences were submitted to the MEME website (https://meme-suite.org/meme/tools/meme (accessed on 23 July 2021)) [49]. The gene structure and protein motifs were visualized in TBtools (version 0.1098765) software [50].

### 4.4. Analysis of Gene Collinearity and Duplication Events

The BlastP (E < 1 × 10 − 10, top 5 matches, and m8 format output) was performed between the different species and subgenomes, and the MCScanX (with default parameter) was used to search all collinearity gene pairs and finally visualized in Circos (version 0.69-9) [51,52,53]. The duplication event and the related gene pairs within each *Gossypium* species and subgenome were identified and classified by the duplicate_gene_classifier program in MCScanX and Dupgen_finder, respectively [53,54].

### 4.5. The Calculation of Selective Pressure

The protein and coding sequences of detected gene pairs were aligned by performing MAFFT software, respectively [45]. The aligned results were further formatted into an AXT format using the ParaAT pipeline [55]. Next, the Kaks_calculator (version 2.0) was employed to calculate the synonymous rate (Ks), nonsynonymous rate (Ka), and their ratio (Ka/Ks) of each gene pair [56].

### 4.6. Analysis of Cis-Elements in the Promoter of GhDUF4228 Genes

The *cis*-elements in the 2000 bp upstream genomic DNA sequences were submitted to the PlantCARE website (http://bioinformatics.psb.ugent.be/webtools/plantcare/html (accessed on 1 August 2021)) to predict the *cis*-elements [57].

### 4.7. Expression Profile of GhDUF4228 Genes in Different Tissues

The transcriptome data in a previous study (Accessions: PRJNA490626) were obtained from the SRA database [16]. The raw RNA-seq reads were filtered by Trimmomatic (version 0.3.9) with the default parameter [58]. Next, the FPKM (Fragments Per Kilobase of transcript per Million mapped reads) value was generated by performing the HISAT (version 2.1.0) + StringTie (version 2.0) pipeline, and the ZJU2.1 version genome was set as the reference genome [16,59]. The final expression levels were shown as log2 (FPKM + 1).

### 4.8. Plant Materials, Abiotic Stress Treatments, and qRT-PCR Analysis

The seeds of *G. hirsutum* (TM-1) were grown in a greenhouse with a light/dark cycle: 27 °C 16 h/22 °C 8 h. Salt, drought, ABA, and MeJA treatments were carried out, respectively, when cotton stretched out the third true leaf. The method of salt and drought treatment is to soak the roots of cotton seedlings in 200 mM NaCl and 30% PEG6000 solution [60], respectively. ABA and MeJA were treated by the spraying method, 200 mM ABA and 100 mM MeJA solution were sprayed evenly on cotton seedling leaves [60,61]. A total of 0.1 g of leaves was treated to 0, 1, 3, 6, 9, 12, and 24 h, the collected samples were transferred to liquid nitrogen immediately, then preserved at −80 °C [62]. Total RNA from the samples was extracted using the polysaccharide and polyphenol-rich RNAprep Pure Plant Kit (TIANGEN, Beijing, China). The extracted RNA was reverse transcribed into cDNA using Prime Script RT kit (TaKaRa Bio, Shiga, Japan) and used as the template. The expression levels of *GhDUF4228* genes under different treatment conditions were detected by qRT-PCR (Promega, Madison, WI, USA). Design fluorescent quantitative specific primers from the website (https://www.genscript.com/tools/real-time-pcr-taqman-primer-design-tool (accessed on 9 August 2021)) using ABI 7500 real-time PCR system (Applied Biosystems, Waltham, CA, USA) to perform PCR experiments, and all samples set up 3 repetitions. The qRT-PCR primers for *GhDUF4228* genes are listed in Table S6, and *GhACTIN* was used as a component expression control in qRT-PCR experiments. The results were calculated using the 2^−ΔΔCt^ relative quantification method [63,64].

### 4.9. Virus-Induced Gene Silencing of Cotton GhDUF4228-67

VIGS is a gene transcription technology developed according to the defense mechanism of plants against RNA viruses to characterize plant gene function, and the technology is based on the Tobacco Rattle Virus (TRV) vector [65]. The TRV system includes two vectors, pTRV1 (pYL192) and pTRV2 (pYL156), respectively. The pTRV2 vector can insert the target gene fragment to silence the target gene. A 300 bp silenced fragment in the *GhDUF4228-67* coding sequence was designed using the website Sol Genomics Network (https://vigs.solgenomics.net/ (accessed on 8 October 2021)), and primers were designed with Oligo7 (version 7.60) software (Table S6) [66]. The silenced fragment was PCR-amplified and ligated into the pTRV2 vector to generate the pTRV2: *GhDUF4228-67* construct, and the recombinant vector was transformed into *Agrobacterium tumefaciens* strain LBA4404. *Agrobacterium* cultures of pTRV2: *GhDUF4228-67*, pTRV2: *00* (negative control), pTRV2: *GhPDS* (positive control), and pTRV1 (pYL192) were collected by centrifugation, using buffer containing 10 mM MgCl_2_, 10 mM MES, and 200 μM acetosyringone. The bacteria were resuspended to OD_600_ = 1.5. After standing in the dark at 25 °C for 3 h, the *Agrobacterium* resuspended solution containing pTRV2: *GhDUF4228-67*, pTRV2: *00*, pTRV2: *GhPDS* was mixed with the *Agrobacterium* resuspended solution containing pTRV1 according to the ratio of 1: 1. Each of the three mixed resuspensions was infiltrated into 8-day-old cotton cotyledons using a syringe [67]. When the leaves of the pTRV2: *GhPDS* plants showed an albino phenotype, the leaves of the pTRV2: *GhDUF4228-67* and pTRV2: *00* plants were taken to extract RNA. When cotton grew to the three-leaf stage, the roots of positive plants were soaked in 200 mM NaCl solution, and deionized water was used as a control group. Changes in RWC of leaves before and after treatment were detected, and proline content, POD activity, and MDA content were extracted and identified according to standard methods (Solarbio, Beijing, China).

## 5. Conclusions

DUF4228 proteins play an essential role in plant response to abiotic stress. In this study, a total of 308 DUF4228 proteins were identified in four cotton species, which can be divided into five subfamilies. Moreover, we further found that WGD events and allopolyploidization were the main driving forces for the expansion of the DUF4228 gene family, and the structure, conserved motifs, and DUF4228 genes were relatively conserved during many rounds of duplication events. Additionally, the qRT-PCR results implied that the *GhDUF4228* genes might be associated with cotton response to abiotic stress and be regulated by exogenous hormones. Furthermore, based on the phenotypic and physiological indicators after salt stress, we speculated that *GhDUF4228-67* might be a positive regulator in cotton response to salt stress. These results have not only laid a solid foundation for further research on the function of DUF4228 proteins, but they have also provided materials for cotton breeding under salt tolerance.

## Data Availability

Not applicable.

## Supplementary Materials

The following supporting information can be downloaded at: https://www.mdpi.com/article/10.3390/ijms232113542/s1. Figure S1: Domain analysis of GhDUF4228 proteins. Conserved domains of GhDUF4228 proteins were identified using SMART. The domain of DUF4228 is represented by a green box. Figure S2: *Cis*-element analysis of the *GhDUF4228* gene’s promoter. The promoter sequences of 103 *GhDUF4228* genes were analyzed by the PlantCARE website (https://bioinformatics.psb.ugent.be/webtools/plantcare/html/ (accessed on 1 August 2021)). The 103 *GhDUF4228* genes are divided into five subfamilies, various *cis*-elements are represented by different colors, and the icons are located in the upper right corner. The tick marks at the bottom are used to infer the length of the upstream sequence from the translation start site. Figure S3: The expression of *DUF4228* genes under salt and PEG stresses in transcript profiling. Table S1: Basic information and biophysical properties of the predicted DUF4228 proteins in cotton. Table S2: The information of identified motifs in GhDUF4228 proteins. Table S3: The duplication types of DUF4228 proteins in cotton. Table S4: The Ka and Ks values for homologous gene pairs in cotton. Table S5: The information of species and their genome used for identification of DUF4228 proteins in this study. Table S6: List of primers used for qRT-PCR analysis and virus-induced gene silencing (VIGS) experiments.
