# Supplementary figures and images for: A Comprehensive Analysis of the DUF4228 Gene Family in Gossypium Reveals the Role of GhDUF4228-67 in Salt Tolerance
Source: Int J Mol Sci. 2022 Nov 4;23(21):13542. doi: 10.3390/ijms232113542 (PMC9655460; doi:10.3390/ijms232113542)

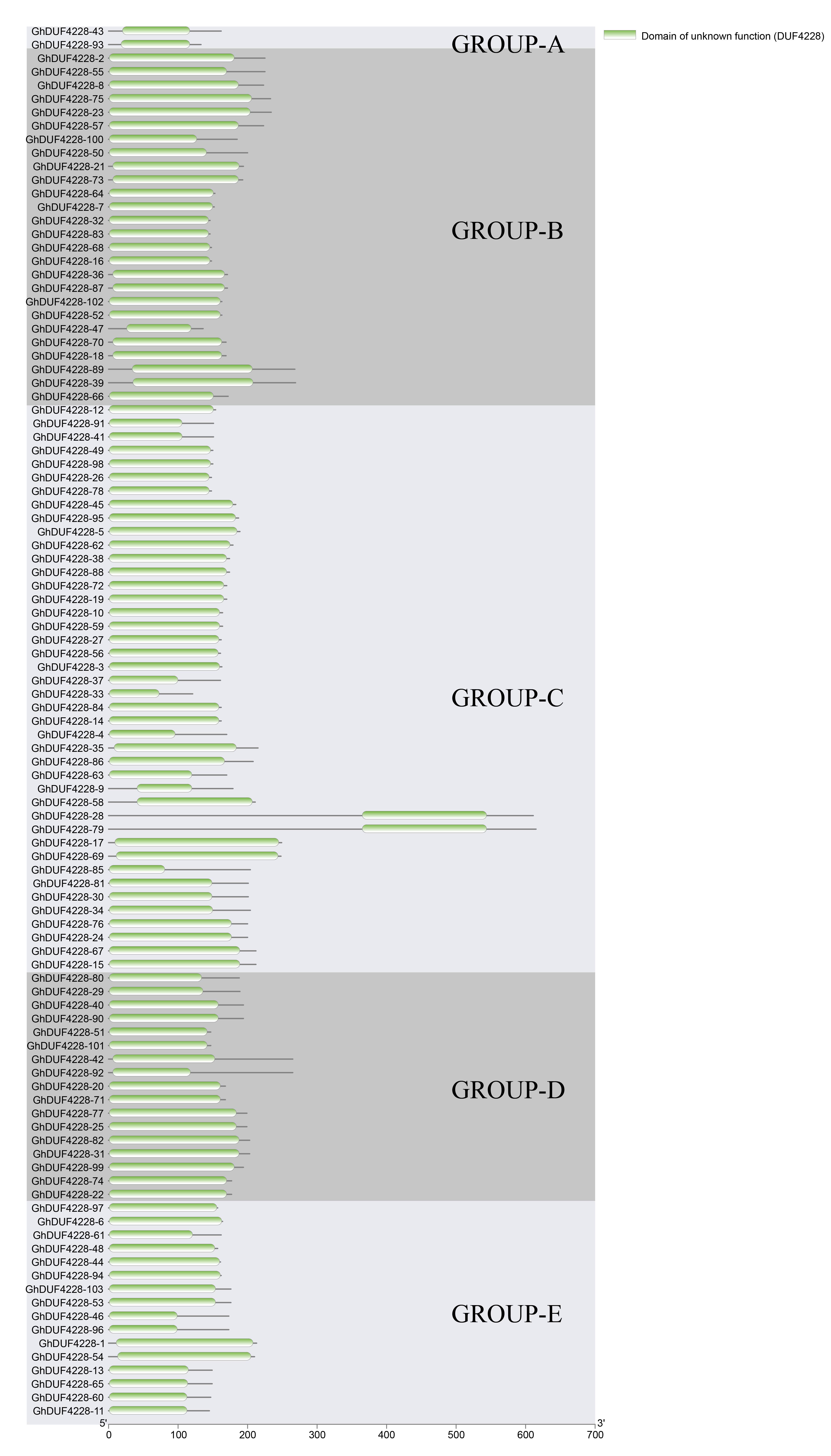

Supplement: Supplementary file 1 [file ijms-23-13542-s001.zip › Figure S1.jpg]

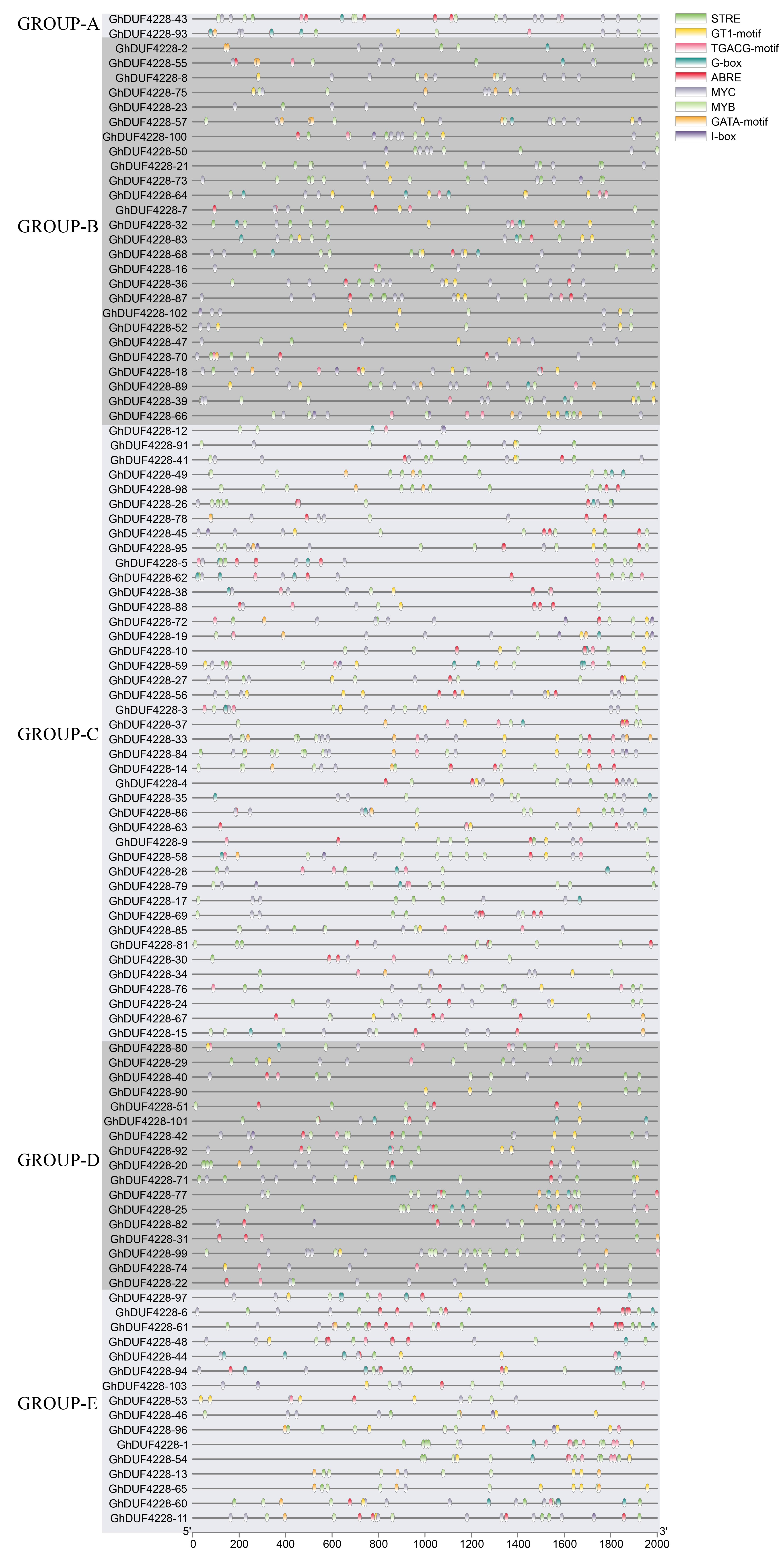

Supplement: Supplementary file 1 [file ijms-23-13542-s001.zip › Figure S2.jpg]

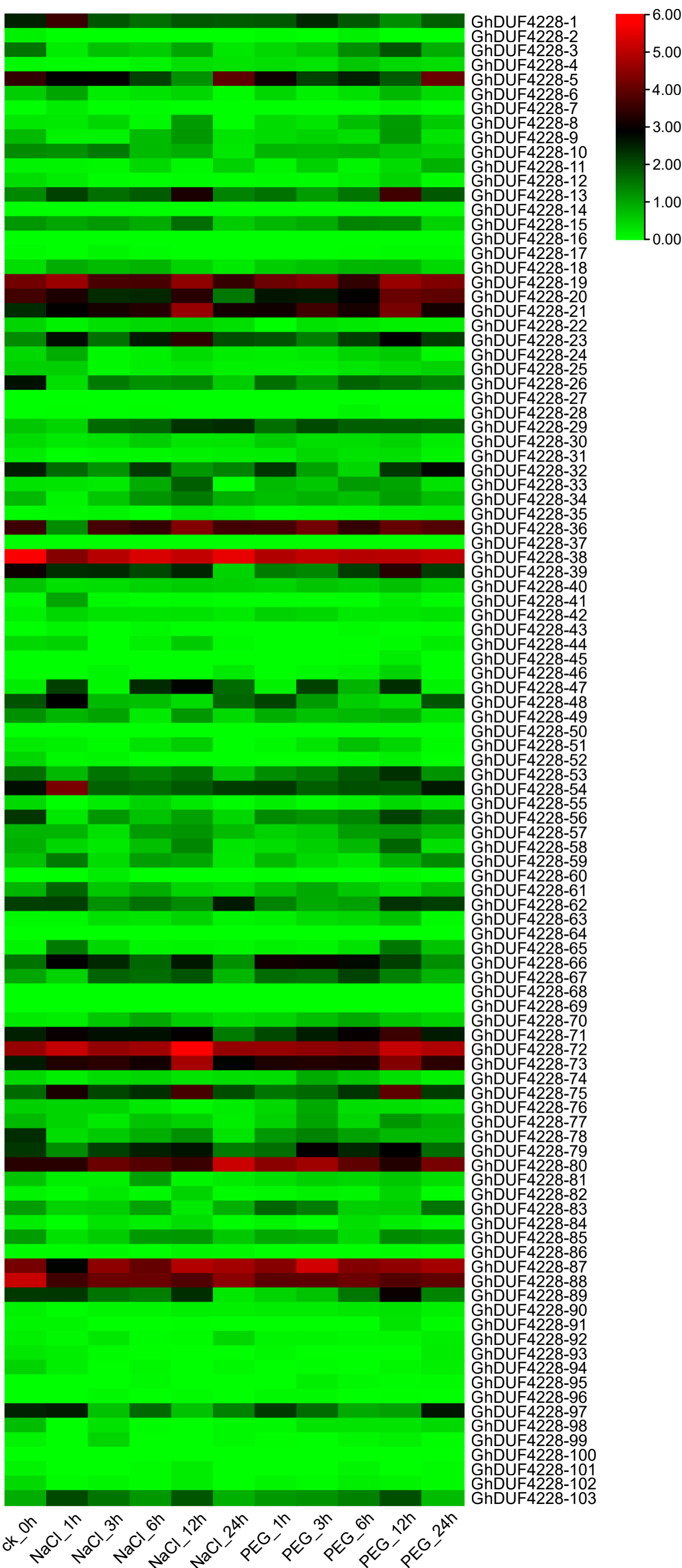

Supplement: Supplementary file 1 [file ijms-23-13542-s001.zip › Figure S3.pdf]
